# Dietary Fermented Chicory Root Waste Modulates Growth, Chemical Composition, Lipid Metabolism, and Intestinal Barrier Pathways in Common Carp (*Cyprinus carpio* L.) Fed With High-Fat Diets

**DOI:** 10.1155/anu/2234393

**Published:** 2025-10-26

**Authors:** Jianing Gu, Xue Tian, Tiantian Wang, Shuxia An, Boya Yang, Zhenyi Huang, Xulu Chang, Guokun Yang, Shikun Feng, Xindang Zhang, Yanmin Zhang, Mohammed A. E. Naiel, Xiaolin Meng

**Affiliations:** ^1^College of Fisheries, Henan Normal University, Xinxiang 453007, China; ^2^Engineering Technology Research Center of Henan Province for Aquatic Animal Cultivation, Henan Normal University, Xinxiang 453007, China; ^3^Animal Production Department, Zagazig University, Zagazig 44519, Egypt

**Keywords:** agro-waste, fermentation, lipid pathway, microbiota, sustainability

## Abstract

The current trial sought to assess the impact of fermented chicory root waste (FCRW) dietary administration on growth, lipid metabolism, chemical composition, and intestinal barrier pathway in common carp (*Cyprinus carpio* L.). Firstly, a single-factor experiment followed by an orthogonal test indicated the optimum factors, such as 30°C for 36 h, a 10% inoculation amount, and a 65% solid–liquid ratio for producing FCRW containing a 12.24% protein. A total of 180 common carp, with an average initial weight of 26.99 ± 4.04 g, were randomly allocated into 12 tanks, with each tank housing 15 individuals. The initial group functioned as the control group (CG) and was provided with a basal diet; meanwhile, the remaining three groups were fed high-fat (HF) diets supplemented with various levels of FCRW, 0%, 5%, and 15% for HF, HF-L, and HF-H, respectively. The feeding trial was prolonged to 56 days. The results of the feeding trial demonstrated that the fish group receiving an HF diet supplemented with a greater proportion of FCRW (15%) exhibited superior growth and feed efficiency. Both 5% and 15% FCRW significantly reduced VSI and HSI, while 15% FCRW increased whole-body crude protein and decreased body/liver lipids. FCRW supplementation also lowered serum/liver triglycerides and serum LDL-C. Additionally, all FCRW levels enhanced antioxidant markers (MDA, AKP, CAT, superoxide dismutase [SOD]) and innate immunity (LZM). Histology showed reduced hepatocyte vacuolation and lipid droplets. Crucially, 15% FCRW upregulated lipolysis genes (*lpl*, *hsl*, *ppar-α*) and downregulated lipogenesis genes (*acc-α*, *fas*, *ppar-γ*). Regarding intestinal structural integrity, FCRW improved intestinal morphology and upregulated barrier genes (*occludin*, *claudin-3*, *zo-1*). It suppressed proinflammatory cytokines (*il-1β*, *il-6*) and activated anti-inflammatory pathways (*il-10*, *tgf-β*, *tlr4*, *nf-κb*). Gut microbiota analysis revealed increased beneficial bacteria (e.g., *Firmicutes*).

## 1. Introduction

Recently, high-fat (HF) diets have been widely applied in fish feed to meet fish's energy requirements and safe protein for growth [1]. In addition to their nutritional advantages for aquatic organisms, fats can enhance protein efficiency, serve as an additional energy source or partially replace protein, boost growth, stimulate reproductive traits, and reduce feed and total production costs. However, under extensive production systems, incorporating HF levels in fish feed can lead to undesirable consequences, such as lipid metabolic disorders, excessive fat accumulation, induced inflammation, suppressed immune responses, and generated endoplasmic reticulum stress in various fish species [2, 3]. For example, an HF diet significantly increased serum LDL-C and liver T-CHO levels in the spotted perch (*Lateolabrax maculatus*), accompanied by downregulation of fatty acid oxidation genes and upregulation of lipogenic genes, collectively promoting lipid deposition [4]. After zebrafish (*Danio rerio*) consumed HF feed, it exhibited pronounced hepatic steatosis with elevated TG, TC, and LDL-C concentrations, alongside upregulated expression of lipid synthesis regulators [5]. Substituting 45% fish oil with terrestrial lipids in gilthead seabream (*Sparus aurata*) suppressed growth performance and activated the nf-κb pathway, triggering the release of proinflammatory cytokines and establishing chronic inflammation [6].

Many dietary supplements, including minerals, vitamins, organic compounds, and various plant-derived byproducts, have recently been shown to regulate lipid metabolism disorders [2]. Therefore, these supplements can help reduce fat accumulation, alleviate hepatic steatosis, and diminish inflammatory responses in fish-fed HF diets. Chicory (*Cichorium intybus* L.), native to Europe and North America, contains functional polysaccharides, including prebiotic inulin and dietary fibers. Inulin, a group of naturally occurring polysaccharides in many plant parts, is most commonly extracted from chicory [7]. Researchers have found that fresh chicory roots contain, by dry weight, 68% inulin, along with 14% sucrose, 5% cellulose, 6% protein, 4% ash, and 3% other compounds [8]. Inulin has been shown to offer several benefits, including improving intestinal hemostasis, enhancing immunity, optimizing feed utilization, and promoting growth [9]. Recent studies on *Eriocheir sinensis* [10], *Oreochromis niloticus* [11], and *Oncorhynchus mykiss* [12] indicate that inulin can significantly enhance the digestive function of aquatic species. Additionally, inulin is instrumental in the regulation of lipid metabolism, enhancing immune function, increasing antioxidant capacity, and the improvement of hepatic and intestinal health, all of which contribute to the growth of farmed fish [13]. Therefore, inulin can potentially mitigate the adverse effects of HF diets in aquaculture, highlighting the need for further research.

Fermentation is an inexpensive and easily applicable method for enhancing the nutritional value of various plant-based ingredients or waste while reducing their antinutritional factors [14]. Under controlled conditions, natural or synthetic probiotics are used during fermentation, producing a new feed resource rich in protein, probiotics, and their exerted metabolites [15]. Common probiotics used in fermented feed include *Lactobacillus* [16], *Bacillus* [17], *Saccharomyces* [18], and *Aspergillus* [19]. Compared to conventional ingredients, fermented plant-based ingredients have shown significant potential for enhancing growth, disease resistance, and immunity in various fish species. However, research on their application in aquatic animals is still required, particularly focusing on fermentable plant waste [20] and using certain fermented plant resources as alternatives to fish meal [21] to sustain the fish feed industry.

Common carp (*Cyprinus carpio* L.) play a significant economic role in the freshwater aquaculture industry due to their remarkable adaptability to various environmental conditions [22]. However, intensive aquaculture systems that utilize HF diets for carp have resulted in several undesirable effects, including inhibited growth, excessive fat accumulation, and health deterioration [23]. Therefore, this trial was designed to evaluate the protective potential and effectiveness of fermented chicory root waste (FCRW) when incorporated into HF diets, focusing on the growth performance, chemical analysis, lipid metabolism regulation pathways, and intestinal barrier integrity of common carp juveniles.

## 2. Materials and Methods

### 2.1. Materials and Reagents

Chicory root waste was obtained from Inner Mongolia Weinihao Co., Ltd. Meanwhile, *Lactococcus lactis* and *Clostridium butyricum* were sourced from Henan Jinbaihe Biotechnology Co., Ltd., while *Aspergillus niger* was isolated and characterized entirely within the laboratory. The analytical grade reagents, such as Reinforced Clostridium medium, MRS agar medium, and potato dextrose agar medium, were purchased from Beijing Solarbio Science & Technology Co., Ltd.

### 2.2. The Examination of Fermentation Variables

The first single-factor trial was designed to determine the optimal levels for four critical variables (fermentation duration, temperature, bacterial inoculation quantity, and solid–liquid ratio). Initially, five fermentation durations (24, 36, 48, 60, and 72 h) were evaluated while keeping the other parameters constant [24]. After identifying 36 h as the optimal fermentation period, five fermentation temperatures (28, 30, 32, 34, and 36°C) were tested while preserving the bacterial inoculation level at 5% and fixing the solid–liquid ratio at 65%. Next, varying bacterial inoculation amounts (1%, 2.5%, 5%, 7.5%, and 10%) were assessed while keeping the fermentation period at 36 h and the temperature at 30°C with the fixed solid–liquid ratio at 65%. Finally, the optimal solid–liquid ratios were estimated by applying several levels (55%, 60%, 65%, 70%, and 75%) while maintaining the fermentation period at 36 h, the temperature at 30°C, and the bacterial inoculation level constant at 10%. In conclusion, the results from these single-factor experiments established the optimal levels for each variable. Subsequently, an orthogonal experiment was applied to evaluate the efficiency of all tested factors at their levels (Table 1).

### 2.3. Diet Preparation

After fermenting chicory root waste under optimal conditions, four formulated diets containing 37% crude protein were prepared, with two varying levels of crude lipid (6% and 12%), ensuring that all diets were isonitrogenous and met fish requirements. The tested diets were prepared with varying concentrations of FCRW at 0%, 5%, and 15%. Initially, the ingredients were processed through an 80-mesh screen, and the resulting material was thoroughly mixed with fish meal, wheat flour, soybean oil, and water. This mixture was then subjected to a pelleting process. The completed feed was subjected to drying in a controlled, cool environment and subsequently stored at a temperature of −20°C for future utilization. The comprehensive composition of the experimental diets is detailed in Table 2.

### 2.4. Fish Welfare, Growth Trial, and Rearing Conditions

All research involving animals is carried out in accordance with the guidelines established by the Animal Ethics Committee. For the purposes of this study, juvenile common carp were acquired from a hatchery located in Henan Province. The initial body weight of the acquired fish was recorded as 26.99 ± 4.04 g. The fish were housed in 12 tanks, with each tank containing 15 individuals, and were subjected to a domestication period of 1 month. Feeding was conducted at designated intervals of 8:00 a.m., 11:30 a.m., and 5:30 p.m. each day.

The handling and transportation of experimental fish adhered to animal ethical guidelines in compliance with the protocols established by the Animal Ethics Committee at Henan Normal University. The initial group functioned as the control group (CG) and was provided with a basal diet. In contrast, the other three groups were administered HF diets supplemented with varying amounts of FCRW, as detailed below: HF (HF diet group, diets containing a high amount of fat at 12%); HF-L (HF diet supplemented with a low level of FCRW at 5%); and HF-H (HF diet supplemented with a high level of FCRW at 15%). Feeding was afforded three times a day (8:00 a.m., 11:30 a.m., and 5:30 p.m.); and the feed allowances were adjusted biweekly to 3% of fish's live body weight. The feeding trial was conducted over an 8-week period. Meanwhile, water quality measurements were maintained within desirable conditions to support fish growth as follows: water temperature was observed to fluctuate between 25 and 27°C, while the pH was 6.5–7.8, and dissolved oxygen levels varied from 6 to 8 mg/L.

### 2.5. Sample Collection

By the end of the feeding trial period, the fish underwent a fasting period lasting 12 h prior to the collection of samples. First, the fish were anesthetized using MS-222 (MCE, USA) at a concentration of 10 mg L^−1^. Then, all measurements for body weight and visceral weight of the experimental specimens were systematically recorded using an electronic scale. To evaluate whole-body composition, three fish were collected from each tank. Blood samples were obtained from the caudal vein of six fish per tank, and serum was obtained by centrifuging the samples at 3500 × *g* for a duration of 10 min at a temperature of 4°C to assess lipid profile-related parameters. Additionally, the same fish from which blood was collected were used to dissect liver and intestinal samples, which were then weighed and preserved to investigate liver lipid metabolism, intestinal antioxidant activity, the relative expression of mRNA, and the intestinal microbial population. Four fish were randomly chosen from each tank for histological evaluation, and their liver and midguts were collected and preserved in a 3% paraformaldehyde solution. Whole intestines from six fish per tank were collected and placed in liquid nitrogen for gut microbiota assessment.

### 2.6. Chemical Analysis Procedure

The analysis of moisture content, crude protein, crude lipid, and crude ash in the formulated feed, liver, and whole fish body was performed in accordance with the procedures established by AOAC [25].

### 2.7. Lipid Profile Related Indicators and Intestinal Innate Immunity and Antioxidant Capacity

The lipid profile-related parameters assessed included TG, LDL-C, and HDL-C. Additionally, indices of intestinal antioxidant activity were measured, specifically MDA, superoxide dismutase (SOD), CAT, AKP, and innate immunity indices such as LZM. All measurements were conducted using commercial kits obtained from the Nanjing Jiancheng Bioengineering Institute (Nanjing, China), following the instrument guidelines for enzyme marker analysis.

### 2.8. Histological and Histomorphometrical Examination

Specimens of the midgut and liver were initially preserved in a 4% paraformaldehyde solution, subsequently subjected to dehydration using ethanol, and ultimately embedded in paraffin. The paraffin sections of the midgut and liver samples were sliced into 6 μm sections and subjected to staining with the hematoxylin–eosin (H&E) staining kit (Solarbio, China). For frozen liver samples, the oil red technique (Solarbio, China) was employed to stain lipid cells in 15 µm thick sections for 7 min, which were prepared using a Leica RM 2250 cryostat (Leica Microsystems Nussloch GmbH, Nussloch, Germany). Afterward, a hematoxylin counterstain was applied for 3 minutes to stain the nuclei, and the sections were rinsed three times with distilled water. Periodic acid-Schiff (PAS) staining techniques were utilized to detect neutral and acidic mucins. The histomorphometric indices of the midgut were assessed using ImageJ v1.53e software. Villus height measurement: For each histological section, five structurally intact villi were selected. The vertical distance from the crypt-villus junction to the villus apex was measured. Muscle layer thickness measurement: For each section, thickness was measured perpendicular to the muscle fiber orientation at five evenly distributed points.

### 2.9. RNA Extraction and Real-Time Quantitative PCR (qPCR)

Total RNA was isolated from the entire liver and intestinal tract using RNAiso Plus (TaKaRa, Japan). The concentration of the RNA was quantified with a NanoDrop 2000, after which reverse transcription was performed to synthesize cDNA. The expression levels of several genes were assessed, specifically those associated with hepatic lipid metabolism (including *lpl*, *hsl*, *fas*, *acc-α*, *ppar-α*, and *ppar-γ*), intestinal inflammatory mediators (such as *il-1β*, *il-6*, *il-10*, *tgf-β*, *tlr4*, and *nf-κb*), and genes related to the integrity of the intestinal physical barrier (namely *zo-1*, *claudin-3*, and *occludin*). Primers for these genes were designed using Premier 5.0 (Table 3). In this investigation, the relative expression of mRNA was determined using the 2^-ΔΔCt^ method, with standardization performed using *β-actin* ribosomal mRNA as a housekeeping gene.

### 2.10. Intestinal Microbial Population

Genomic DNA was isolated from intestinal samples utilizing a DNA extraction kit. Next, the V3-V4 region of the 16S rRNA gene was amplified with specific primers. All data analyses related to 16S rRNA high-throughput sequencing were conducted on the Nanjing Paisonol data analysis cloud platform. To evaluate the evenness and diversity of the gut microbiota, we calculated alpha diversity metrics, ACE, Chao1, Shannon, and Simpson indices, using QIIME. Additionally, rarefaction curves were generated to assess the adequacy of sequencing depth. Finally, the composition of the microbial communities was analyzed by employing heatmaps, concentrating on both the phylum and genus taxonomic levels.

### 2.11. Statistical Analysis

All collected data were subjected to assessments of normality and homogeneity through the application of the Shapiro–Wilk test. Then, the data analysis was performed using Excel 2021, while graphical representations were created with GraphPad Prism version 9.5.1. Statistical differences among the studied variables during fermentation were analyzed using a *t*-test following an orthogonal contrast test. After a one-way ANOVA analysis using SPSS statistical software version 22.0 (IBM, USA), Duncan's multiple range test was also applied to evaluate differences between treated and nontreated groups. All data are presented as mean ± SEM, with a significance threshold set at *p* < 0.05, indicating statistical significance.

## 3. Results

### 3.1. Single-Factor Fermentation Optimization

**Figure 1:**
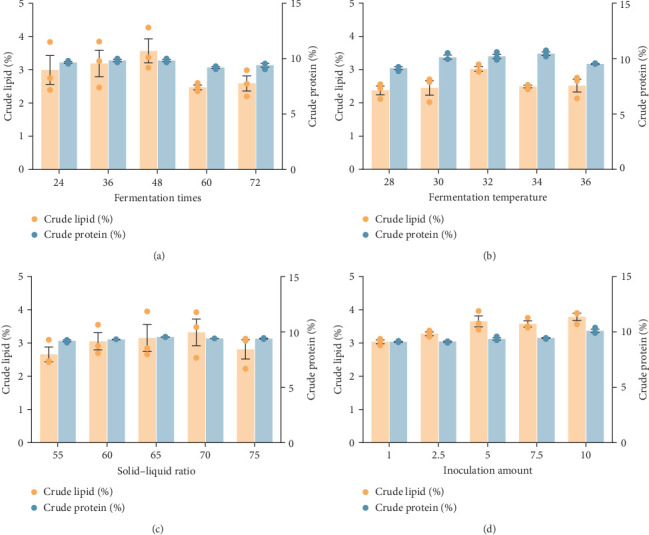
The single-factor experiment plots show the content of crude lipid and crude protein: (A) fermentation times (h); (B) fermentation temperature (°C); (C) solid–liquid ratio (%); and (D) inoculation amount (*n* = 3).

Figure 1 represents data from single-factor trials to estimate the influences of fermentation duration, temperature, inoculation amount, and solid–liquid ratio on the fermentation of chicory root waste. Significant variations were observed among the different parameters within each trial group. The accuracy of the subsequent orthogonal comparison and the presented data indicated the optimal results as follows: fermentation duration of 24, 36, and 48 h; fermentation temperatures of 30, 32, and 34°C; inoculation amounts of 5%, 7.5%, and 10%; and solid–liquid ratios of 60%, 65%, and 70%.

The orthogonal analysis of the data in Table 4 indicates that several factors influence the crude protein content of FCRW. The order of these factors is as follows: *A* > *C* > *D* > *B* (Table 1). Furthermore, Table 5 shows that the primary factors significantly affecting the crude protein content of FCRW during the fermentation process are fermentation temperature, inoculation amount, and solid–liquid ratio, whereas fermentation time has no significant impact. Herein, the optimal fermentation conditions were identified as A3, B2, C1, and D2, which correspond to a fermentation temperature of 30°C, a duration of 36 h, an inoculation amount of a mixed starter of *L. lactis*, *C. butyricum*, and *A. niger* (1:1:1 ratio) at 10%, and a solid–liquid ratio of 65%.

After conducting the orthogonal test, a verification process was carried out to confirm the reliability of the obtained order (A3-B2-C1-D2). The verification results were similar to the optimal outcome observed in the orthogonal test (Table 4). Specifically, our study's verification indicated that a protein level of 12.24% was attained under the specified conditions: a fermentation temperature of 30°C for 36 h, an inoculation amount of 10% (a mixed starter of *L. lactis*, *C. butyricum*, and *A. niger* in a ratio of 1:1:1), and a solid–liquid ratio of 65%.

### 3.2. Growth, Feed Efficiency, and Whole Fish Proximate Composition

As shown in Table 6, compared with the HF group, final body weight (FBW), weight gain ratio (WGR), and specific growth rate (SGR) all indicate a remarkable improvement (*p* < 0.05), along with a notable reduction in feed conversion ratio (FCR) values. The best growth performance and feed efficiency measurements were observed in the fish group fed an HF diet supplemented with a higher amount of FCRW (15%). Meanwhile, both incorporation levels of FCRW (5% and 15%) in fish diets containing high fat significantly (*p* < 0.05) reduced VSI and HSI levels compared to the fish group fed an HF diet without any treatment. The fish group that received a diet high in fat and was administered a low level of FCRW (5%) exhibited the lowest value of the VSI. Conversely, the group of fish that was provided with an HF diet supplemented with a higher quantity of FCRW (15%) demonstrated the lowest value of the HSI. Conversely, the condition factor (*K*) values showed no significant differences among all tested groups.

According to the data in Table 7, which presents significant (*p* < 0.05) effects of dietary FCRW on the whole-body composition of fish fed an HF diet compared to the HF group. The current results indicate significant (*p* < 0.05) increases in crude protein content in the whole body of fish that were given diets containing high fat and supplemented with a high amount of FCRW (15%). Meanwhile, the lipid content in the fish liver and their whole bodies decreased significantly (*p* < 0.05) with increasing levels of FCRW incorporated into the diet compared to the HF group. The lowest lipid content was recorded in the fish group fed HF diets and supplemented with a high level of FCRW (15%).

### 3.3. Lipid Profile-Related Measurements

The TG content in both serum and liver, and the LDL-C levels in serum, showed a significant decline (*p* < 0.05, Table 8) with the incorporation of FCRW into HF fish feed compared to the HF group. Specifically, the lowest TG and LDL-C levels in serum were observed in the HF-H group. Furthermore, the liver TG content was lowest with the HF-L group, and these levels were found to be close to those of the CG group.

### 3.4. Antioxidant and Innate Immune Activities

**Figure 2:**
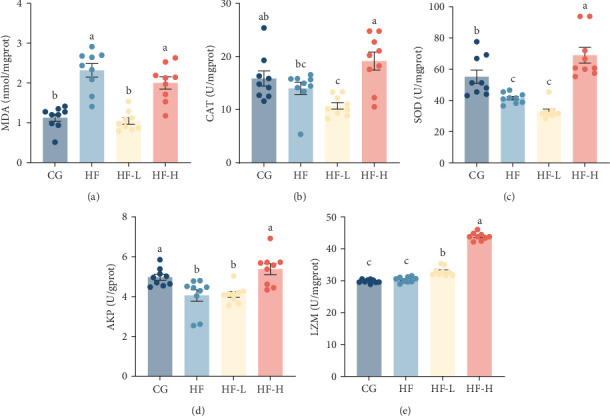
Effects of dietary FCRW supplementation on intestinal innate immunity and antioxidant capacity of common carp fed high-fat diets (*n* = 9; independent *t*-test; means ± SEM). (A) Malondialdehyde (MDA); (B) catalase (CAT); (C) superoxide dismutase (SOD); (D) alkaline phosphatase (AKP); and (E) lysozyme (LZM). Bars with different superscripts show significant differences (*p* < 0.05).

According to the data in Figure 2, which shows the effects of dietary FCRW on intestinal antioxidant and innate immune parameters in common carp-fed HF diets. The incorporation of FCRW at any level led to a significant reduction (*p* < 0.05) in MDA content and notable increases (*p* < 0.05) in both CAT and SOD levels compared to the HF group. The lowest MDA levels were recorded in the fish group fed an HF diet supplemented with a low level of FCRW, while the highest CAT and SOD levels were observed in the fish group receiving HF diets supplemented with a high level of FCRW, compared to the other experimental groups.

The HF-H group exhibited significantly higher (*p* < 0.05) levels of intestinal AKP compared to the HF group. Additionally, LZM activity significantly increased (*p* < 0.05) in all FCRW-supplemented groups compared to the other experimental groups. Specifically, the highest levels of LZM activity were observed in the HF-H group.

### 3.5. Lipid Metabolism

**Figure 3:**
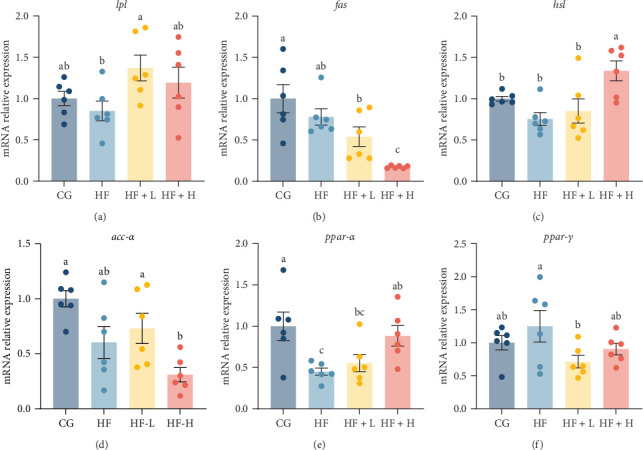
Effects of dietary FCRW supplementation on the expression of lipolysis-related genes ((A) *lpl*, (C) *hsl*, and (E) *ppar-α*) and lipid synthesis-related genes ((B) *fas*, (D) *acc-α*, and (F) *ppar-γ*) in the liver of common carp fed high-fat diets (*n* = 6; independent *t*-test; means ± SEM). Bars with different superscripts show significant differences (*p* < 0.05).

There has been a significant rise in the relative expression levels of all assessed genes associated with lipolysis, including *ppar-α*, *hsl*, and *lpl*, which was observed by incorporating FCRW into fish diets (Figure 3). Notably, the highest upregulation of *lpl* gene expression was detected in the HF-L group compared to the other experimental groups. Meanwhile, the relative expression levels of both *hsl* and *ppar-α* genes were significantly (*p* < 0.05) higher in the HF-H group compared to the HF group. In contrast, the expression levels of genes involved in lipid synthesis, such as *acc-α*, *fas*, and *ppar-γ*, decreased following the incorporation of FCRW. Specifically, a significant downregulation of the *fas* gene was noted after treatment of the HF diets with a high level of FCRW (*p* < 0.05). Similarly, there was a significant downregulation of *ppar-γ* genes in the HF-L group compared to the HF group (*p* < 0.05).

**Figure 4:**
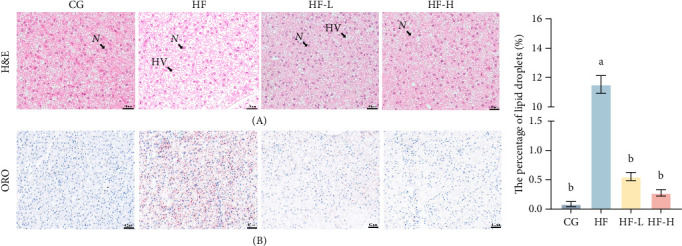
The histological examination of the liver in common carp fed high-fat diets supplemented with various levels of FCRW. (A) H&E staining images (200×), the “*N*” indicates the nucleus and “HV” indicates the lipid vacuoles. (B) Representative ORO-stained pictures (200×). Bars with different superscripts show significant differences (*p* < 0.05).

Figure 4 represents the histological and lipid examination of liver sections stained with two different stains (H&E and Oil Red O). The results from H&E staining indicated that liver cells in the CG group exhibited normal morphological structure, distinct nuclei, well-defined cell boundaries, and fewer lipid vacuoles. In contrast, the HF group displayed a significant number of lipid vacuoles, along with nuclear displacement toward the cell periphery, blurred cell boundaries, and instances of nuclear dissolution or disappearance. Additionally, incorporating varying concentrations of FCRW improved the morphological characteristics of the liver of the fish group that received diets containing high fat, particularly at the highest concentration, where cell structure and morphology remained intact. Furthermore, Oil Red O staining revealed a significantly higher intensity of lipid accumulation in the HF group's liver cells compared to the CG group (*p* < 0.05). Moreover, adding different proportions of FCRW into fish group diets containing high fat remarkably reduced liver lipid accumulation compared with the HF group (*p* < 0.05).

### 3.6. Intestinal Morphology

**Figure 5:**
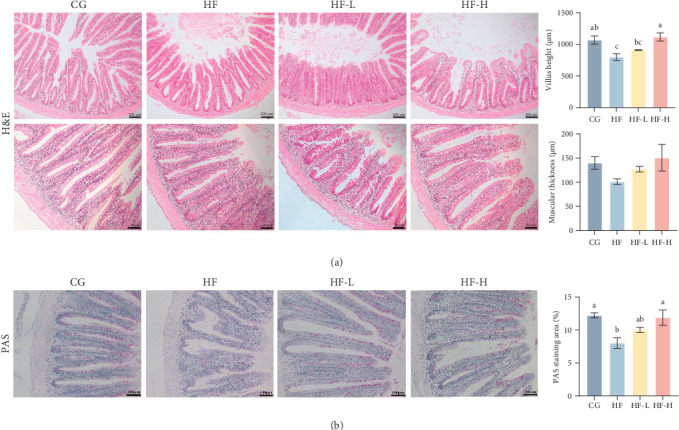
The midgut histological and histomorphometric examination of common carp fed high-fat diets supplemented with various levels of FCRW. (A) H&E staining images, villus height, and muscular thickness. (B) Periodic acid-Schiff stain images and PAS staining area. Bars with different superscripts show significant differences (*p* < 0.05).

The morphological structure of intestinal specimens from common carp fed an HF diet supplemented with various levels of FCRW is shown in Figure 5. The villus height showed significant (*p* < 0.05) suppression in the HF group compared to the CG group. Conversely, supplementing the HF diet with a high level of FCRW significantly (*p* < 0.05) improved the villus height compared to the other experimental groups (Figure 5A). No significant variation was observed in the muscular thickness among the various treatment groups. Furthermore, the intestinal goblet cell count revealed a significantly lower count (*p* < 0.05) compared to the CG group (Figure 5B). Meanwhile, supplementing HF diets with a high level of FCRW significantly increased the goblet cell count compared to the HF group.

### 3.7. Relative Expression of Intestinal Health-Related Genes

**Figure 6:**
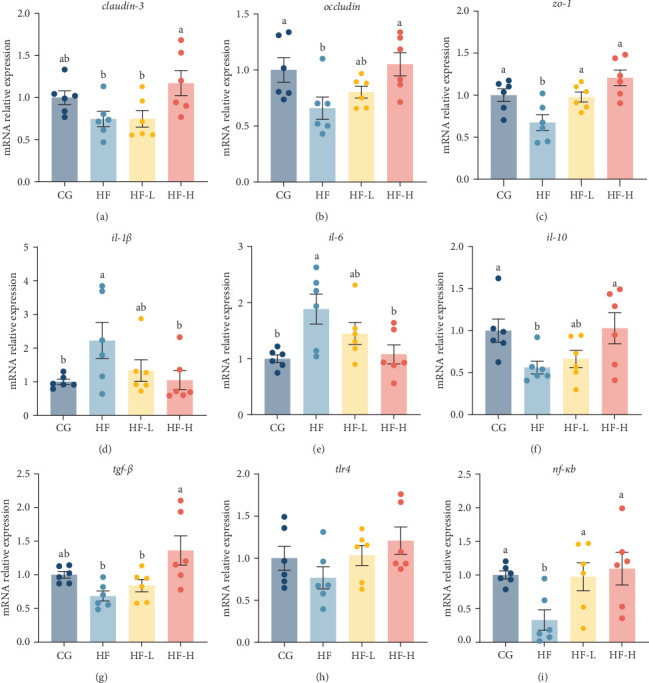
Effects of dietary FCRW supplementation on the expression of (A–C) tight-junction-protein (*claudin-3*, *occludin*, and *zo-1*), (D, E) proinflammatory cytokines (*il-1β* and *il-6*), (F, G) anti-inflammatory cytokines (*il-10* and *tgf-β*) and (H, I) *tlr4*/*nf-κb* pathway-related genes (*tlr4* and *nf-κb*) in the intestine of common carp fed high-fat diets (*n* = 6; independent *t*-test; means ± SEM). Bars with different superscripts show significant differences (*p* < 0.05).

The relative expression levels of intestinal tight junction proteins (*claudin-3*, *occludin*, and *zo-1*) showed significant (*p* < 0.05) upregulation with the supplementation of FCRW in HF fish feed compared to the HF group (Figure 6A–C). However, the proinflammatory cytokines *(il-1β* and *il-6*) showed remarkable downregulation (*p* < 0.05, Figure 6D,E), alongside a significant upregulation (*p* < 0.05, Figure 6F,G) of the anti-inflammatory cytokines (*il-10* and *tgf-β*) under the influence of dietary supplementation with FCRW. The high supplementation level of FCRW resulted in the lowest relative expression levels of *il-1β* and *il-6*, while the same treatment yielded the highest relative expression levels of *il-10* and *tgf-β* compared with the HF group. Finally, the expression levels of pathway-related genes (*tlr4* and *nf-κb*) showed notable improvement when HF diets were treated with various levels of FCRW (Figure 6H,I). Specifically, the high level of FCRW significantly (*p* < 0.05) upregulated both pathway-related genes compared to other experimental groups.

### 3.8. Intestinal Microbiota

**Figure 7:**
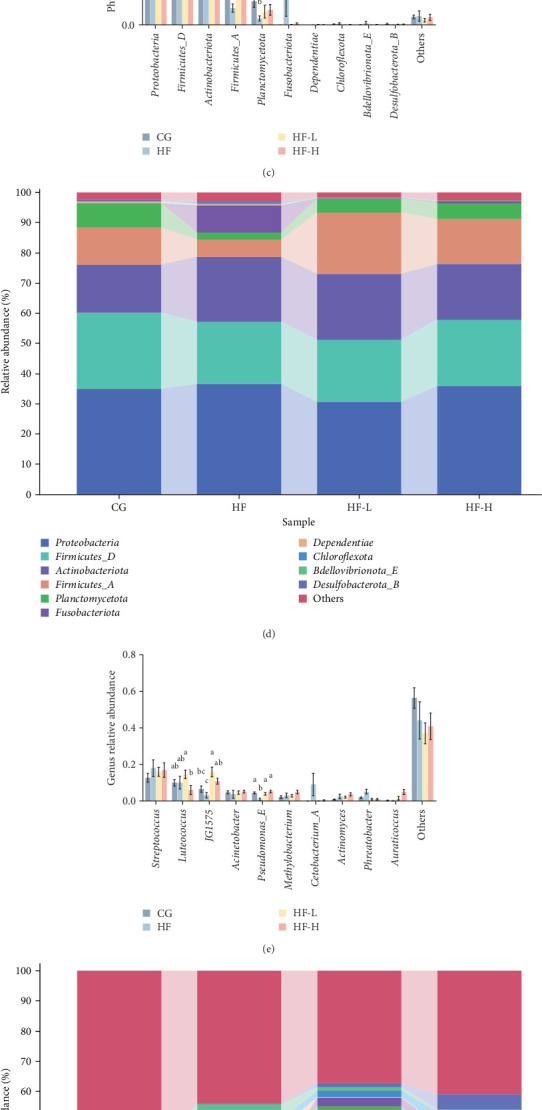
(A) Chao1 indices and (B) Simpson indices. Taxonomy classification of reads from 16S RNA V3–V4 regions of intestinal bacteria at (C) phylum and (E) genus taxonomic levels. Only the top 10 most abundant phyla and genera (based on relative abundance) were shown in the figures, and the relative abundance level of intestinal bacteria at (D) phylum and (F) genus were provided in the figures. Bars with different superscripts show significant differences (*p* < 0.05; *n* = 6; independent *t*-test; means ± SEM).

Figure 7A,B) illustrates the effects of incorporating high fat into fish feed, treated with low or high levels of FCRW, on the Chao1 and Simpson indices in common carp. The Chao1 index data indicate that HF feeding results in a significantly lower total number of intestinal species than the CG group. However, the HF-H group restores the healthy number of intestinal species. In contrast, the Simpson index data showed no significant alterations across all treated and nontreated groups. The most predominant bacterial phyla identified across all groups at the phylum level were *Proteobacteria*, *Firmicutes*, *Actinobacteriota*, and *Planctomycetota*. Notably, there was a significant increase in the abundance of *Planctomycetota* and *Firmicutes* (*p* < 0.05), whereas the abundance of *Proteobacteria* and *Actinobacteriota* decreased with higher levels of FCRW supplementation in fish feed (Figure 7C,D). At the genus level, the dominant genera included *Streptococcus*, *Luteococcus*, *JG1575*, and *Acinetobacter*. More abundances of *JG1575* were observed in fish groups receiving FCRW compared to the HF group (Figure 7 E,F).

## 4. Discussion

Solid-state fermentation is a process in which microbial growth occurs on solid particles without adding free water; however, the moisture in these solid particles is sufficient for microorganism growth [26]. Key factors influencing solid-state fermentation include the nature and size of the substrate, type of microorganism, relative humidity, process duration, and the maintenance of heat and gaseous atmosphere [27]. Therefore, our single-factor experiment, followed by an orthogonal test, identified the optimal conditions: 30°C for 36 h, a 10% inoculation rate, and a 65% solid–liquid ratio. The microorganisms selected for producing fermented FCRW were *Enterococcus faecalis*, *C. butyricum*, and *A. niger*, resulting in a protein content of 12.24%. The results obtained were consistent with those reported in a study by Xu et al. [28], which demonstrated that using *A. niger* in the fermentation process of CRW reduced its crude fiber content. Also, the fermentation process produces protease, cellulase, and other active compounds that enhance the digestion and absorption of various nutrients [29].

Growth performance is an important indicator of oxidative stress damage. However, incorporating a high level (15%) of FCRW into the diet led to significant increases in FBW, WGR, and SGR, while decreasing FCR values. These results confirm that aquatic animals can absorb nutrients from fermented feed more readily than from conventional feed. The improvements in growth and utilization of consumed feed may be attributed to FCRW's ability to enhance intestinal digestive enzyme activity by promoting intestinal peristalsis in fish [30]. Furthermore, chicory has the potential to promote the secretion of substances in the mucosa of the small intestine, as well as in the pancreas and liver, which may improve nutrient digestion and enhance their accessibility at the intestinal brush border [31]. Additionally, research has shown that the primary advantageous effect of a probiotic mixture or chicory is to facilitate favorable changes in the biodiversity of the intestinal microbiota through the selective stimulation of beneficial bacterial populations [32].

Our study results indicated that the lowest VSI value was observed in the fish group fed an HF diet and treated with a low level of FCRW (5%). Herein, the fish group that received an HF diet supplemented with a greater proportion of FCRW (15%) exhibited the lowest HSI value. Therefore, it is speculated that FCRW supplementation in fish feed may help alleviate lipid accumulation in the liver caused by an HF diet. The obtained results are consistent with the findings of Li et al. [33], which suggested that the administration of chicory polysaccharides (50 mg/kg diet) can modify lipid metabolism, improve dyslipidemia, and reduce liver inflammation in HF diet-induced nonalcoholic fatty liver disease (NAFLD) rats.

Our study indicated that the TG content in both serum and liver, and the HDL-C and LDL-C levels in serum, showed a remarkable improvement with incorporating FCRW into HF fish feed compared with the HF group. The findings align with those reported by Huang et al. [34], who reported that an HF diet elevated TG and LDL-C serum levels. Also, previous research indicated that administering chicory water extract to diabetic rats resulted in significant reductions in blood glucose, TG, TC, and LDL-C levels, while HDL-C levels increased significantly, along with an increase in body weight [35]. The improvements in lipid metabolism may be attributed to the efficiency of chicory, primarily due to its inulin component, which might alter the absorption and/or synthesis of cholesterol and increase fecal excretion of lipids, cholesterol, and bile acids [36].

A diet rich in fat has resulted in significant lipid accumulation within the liver, resulting in growth retardation and liver injury in common carp [37]. Our results align with previous studies that indicated dietary inulin supplementation can enhance total body protein levels in *Tachysurus fulvidraco*, promote prolonged protein synthesis, and mitigate the negative effects of ammonia [38].

Current qPCR findings demonstrate a notable elevation in the relative expression levels of all evaluated genes associated with lipolysis, including *ppar-α*, *hsl*, and *lpl*, following the incorporation of FCRW into fish diets. In contrast, the expression levels of lipid synthesis-related genes, such as *acc-α*, *fas*, and *ppar-γ*, decreased after adding FCRW. Thus, understanding the molecular mechanisms that regulate the metabolism and utilization of dietary lipids enhances our comprehension of fish's feeding and growth response when fed specific lipids and treated with certain amounts of FCRW.

In the current study, the activities of CAT and SOD in the intestinal tissues of carp were significantly reduced as well as MDA levels were elevated in fish that were fed an HF diet without any treatment. Conversely, supplementing their diets with a high level of FCRW promoted the activities of both CAT and SOD and suppressed MDA production. The antioxidant properties of FCRW may be attributed to its higher content of flavonoids and phenolic acids, which can influence its oxidative activity [39]. Besides, the hydroxyl groups in phenols facilitate free radical scavenging, thereby reducing the inactivation of antioxidant enzymes that can occur due to the action of these free radicals [40]. Furthermore, reducing free radical content and increasing antioxidant enzyme levels lowers lipid peroxidation in tissues [41].

In the current trial, fish that received an HF diet without any supplementation showed a significant decrease in intestinal AKP and lysozyme activities. However, the addition of fermented chicory root powder resulted in a notable stimulation of both AKP and lysozyme activities, suggesting that incorporating FCRW can enhance the intestinal innate immune defense system and improve the overall health status of fish. These effects may be attributed to the anti-inflammatory properties of chicory roots and their antioxidant mode of action, which includes the ability to inhibit various types of cytokines and stimulate antioxidant enzyme activities that scavenge free radicals [42].

In this study, adding FCRW mitigated the adverse effects of an HF diet on the intestinal barrier. The intestinal villus height in the HF-H group was significantly greater than that in the HF group, leading to improved nutrient absorption and supporting the growth of common carp. Also, our study found that including 15% FCRW significantly enhanced the expression levels of intestinal *zo-1*, *occludin*, and *claudin-3*, thereby strengthening the intestinal physical barrier of common carp. This effect might be due to the highest chicory content from polyphenol compounds, containing over 10% total phenolics, with 71% comprising di-caffeoylquinic acids [43]. These findings align with previous research on other polyphenols that aid in restoring damaged intestinal morphology. Additionally, histopathological damage in fish is often closely associated with oxidative damage [44]. Therefore, our results strongly suggest that FCRW effectively alleviates intestinal oxidative stress induced by an HF diet.

Exposing the intestinal immune barrier to oxidative stress induced several transcription factors that regulate the expression of inflammatory gene pathways [45]. Among these, proinflammatory cytokines serve as primary mediators of the body's defensive system against pathogen invasion and enhance the host's ability to combat pathogens [46]. For example, *il-1β*, *il-10*, and *tgf-β* induce the expression of various proinflammatory genes, leading to elevated triglyceride levels and excessive accumulation of steatohepatitis and fibrosis [47]. In addition, previous findings have demonstrated that Toll-like receptors (TLRs), which fall under the category of pattern recognition receptors, can modulate the immune response by increasing cytokine synthesis, thereby regulating inflammatory responses [48]. The current trial results indicated an upregulation in the expression levels of the *il-1β* and *il-6* genes in the intestines of common carp fed an HF diet. Incorporating FCRW suppressed the expression of *il-1β* and *il-6* genes and restored the regulation of *il-10*, *tgf-β*, *tlr4*, and *nf-κb* gene expression in the intestinal tract. Furthermore, these findings prove that FCRW has the potential to enhance intestinal inflammation regulation and improve the immune response of intestinal tissue through modulation of the TLR4/NF-κB signaling pathway [42].

It has been well documented that HF diets induce changes in gut microbiota, leading to neuronal adaptations that affect central neuropeptides in regulating satiety [49]. Recent reports have shown that HF diets significantly increase the abundance of both *Bacteroidetes* and *Firmicutes* [50]. Consequently, therapeutic modalities aimed at normalizing gut microbiota have been extensively studied, including diets such as the fish diet. The *Firmicutes*/*Bacteroidetes* ratio is a crucial indicator of intestinal flora balance, with an elevated ratio often linked to obesity [51]. Our findings align with prior research indicating that *Fusobacteria*, *Proteobacteria*, *Bacteroides*, *Firmicutes*, and *Actinobacteria* become dominant bacterial groups in fish-fed HF diets' intestinal tracts [52]. By increasing the proportion of *Planctomycetota* and *Firmicutes* in the intestinal cavity, chicory extract helps maintain intestinal barrier function during hyperuricemia and reduces intestinal permeability [53]. Lastly, in regulating intestinal pathogenic bacteria, the high-dose chicory group also significantly reduced the ratio of *Luteococcus*, which can produce virulence factors that damage the intestinal mechanical barrier function [54].

In conclusion, fermented CRW using solid-state fermentation procedures can increase its protein content to 12%. Supplementing common carp HF diets with high levels of FCRW demonstrates a significant growth-promoting effect and reduces the FCR. Additionally, FCRW significantly enhances lipid metabolism-related measurements and chemical body composition, improves the activity of antioxidant enzymes activities in the intestinal tract, and promotes the expression of proteins associated with intestinal tight junctions and anti-inflammatory factors, thereby enhancing immune function. Furthermore, FCRW alters the diversity of intestinal flora, contributing to better intestinal health and mitigating the risks associated with HF diets. Specifically, the optimal supplemental level of FCRW is 15% in HF diets; however, its potential and ideal supplemental levels in fish diets require further investigation.

## Figures and Tables

**Table 1:** Level table of fermentation process factors of chicory root waste.

Factor	*A* (%)	*B* (h)	*C* (°C)	*D* (%)
Level 1	5	24	30	60
Level 2	7.5	36	32	65
Level 3	10	48	34	70

**Table 2:** Formulation and proximate composition of the experimental diets (g/100 g dry matter).

Ingredients	CG	HF	HF-L	HF-H
Wheat bran^a^	15.00	15.00	10.00	0.00
Fermented chicory root waste (FCRW)	0.00	0.00	5.00	15.00
Soybean oil	1.50	6.50	6.50	6.50
Wheat flour	10.60	10.60	10.60	10.60
Soybean meal^a^	25.00	25.00	25.00	25.00
Fish meal^a^	10.00	10.00	10.00	10.00
Pork meal^a^	8.00	8.00	8.00	8.00
Rapeseed meal^a^	15.00	15.00	15.00	15.00
Ca(H_2_PO_4_)_2_^a^	1.00	1.00	1.00	1.00
Vitamin premix^b^	1.00	1.00	1.00	1.00
Mineral premix^c^	1.00	1.00	1.00	1.00
Choline chloride^a^	0.20	0.20	0.20	0.20
Microcrystalline cellulose^a^	11.70	6.70	6.70	6.70
Total	100.00	100.00	100.00	100.00
Proximate composition (g/100 g dry matter basis)				
Crude protein	36.02	37.34	37.26	37.78
Crude lipid	6.35	12.18	13.22	13.67
Crude ash	8.72	10.96	10.32	12.03

^a^Supplied by Tongwei Agricultural Development Co., Ltd. (Sichuan, China). The crude protein and crude lipid levels of the raw material are shown below, respectively. Wheat bran, 16.75% and 5.43%; soybean meal, 50.48% and 1.49%; fish meal, 67.75% and 14.16%; pork meal, 75.21% and 13.96%; rapeseed meal, 42.90% and 3.80%.

^b^Vitamin premix containing the following (mg/kg diet): VA, 264; VB1, 1500; VB, 124; VB2, 1250; VB6, 1100; VC, 2500; VD3, 52.8; VE, 15000; VK, 3325; creatine, 5500; pantothenic acid, 4500; niacin acid, 4000; folic acid, 70; biotin, 125.

^c^Mineral premix containing the following (mg/kg diet): Ca, 330,000; Mg, 45000; P, 105000; Fe, 15000; I, 50; Cu, 350; Zn, 3000; Mn, 1500; Se, 9; Co, 11.

**Table 3:** Real-time PCR primers used for intestinal genes of *Cyprinus carpio* L.

Genes	Forward sequence (5′–3′)	Reserve sequence (5′–3′)	Accession number
*β-actin*	CTGAGAAACGGCTACCATTC	GCCTCGAAAGAGACCTGTATTG	JQ619778.1
*lpl*	CGCTCCATTCACCTGTTCAT	GCTGAGACACATGCCCTTATT	FJ716101.1
*hsl*	ATGATTTGGATGCGCAGACC	AAACGCTCCAGTGCAGTTTG	MF061228.2
*fas*	GACAGGCCGCTATTGCTATT	TGCCGTAAGCTGAGGAAATC	GQ466045.1
*acc-α*	GTCACTGGCGTATGAGGATATT	TCCACCTGTATGGTTCTTTGG	XM_042757417.1
*ppar-α*	GCGTGCTTTGGCTTTGTT	GGGAAAGAGCAGCACGAG	FJ849065.1
*ppar-γ*	GTCAAGTCCGAGATGCACC	GGATGACCTGAGCATTGAAGC	FJ849064.1
*il-1β*	GAGTGAACTGCACCAAACAAC	GTCGGCACTGTCAGAGTAAAT	KC008576
*il-6*	GCCCAGGAACTCACTCTAAAC	GCCCAGGAACTCACTCTAAAC	HQ380208
*il-10*	CTCCGTTCTGCATACAGAGAAA	TCATGACGTGACAGCCATAAG	JX524550
*tgf-β*	TCATGACGTGACAGCCATAAG	GCCACTTTCTTTGTTTGGGAATA	AF056942
*tlr4*	GCCACTTTCTTTGTTTGGGAATA	TGGAGTGTCGCACACATAATAG	HQ731681
*nf-κb*	TGGAGTGTCGCACACATAATAG	CAGTGAAGTCGCGGGAAATA	DQ411314
*zo-1*	AGGAAGTTCTCCCTCGTACTC	AGGAAGTTCTCCCTCGTACTC	KY290394
*claudin-3*	CTCACACAAAGGGTGGAGATAC	CTCACACAAAGGGTGGAGATAC	KF193858
*occludin*	ATGTTGTCCTTCCCGTGATAAG	TCCGTAAGAACCTCCGTAAGA	KF975606

*Note: hsl* = hormone-sensitive triglyceride lipase.

Abbreviations: *acc-α*, acetyl-coa carboxylase-α*; fas*, fatty acid synthesis*; il-1β*, interleukin-1β*; il-6*, interleukin-6*; il-10*, interleukin-10; *lpl*, lipoprotein lipase*; nf-κb*, nuclear factor kappa-b; *ppar-α*, peroxisome proliferators-activated receptor α; *ppar-γ*, peroxisome proliferators-activated receptor γ; *tgf-β*, transforming growth factor β; *tlr4*, Toll-like receptor 4; *zo-1*, zonula occludens 1.

**Table 4:** The effects of several factors on the crude protein content of FCRW during the fermentation process.

Items	*A* (%)	*B* (h)	*C* (°C)	*D* (%)	Crude protein content
Contrast	Unfermented				9.378
1	1	1	1	1	10.251
2	1	2	2	2	9.360
3	1	3	3	3	9.200
4	2	1	2	3	9.457
5	2	2	3	1	10.523
6	2	3	1	2	11.075
7	3	1	3	2	10.694
8	3	2	1	3	10.655
9	3	3	2	1	10.301
*k*_1_	9.5904	10.1044	10.5541	10.3059	—
*k*_2_	10.2831	10.2071	9.7293	10.3845	—
*k*_3_	10.5681	10.1301	10.1581	9.7511	—
*R*-range	0.9777	0.1027	0.8248	0.6333	—
Primary and secondary order: *A* > *C* > *D* > *B*					
Optimal level		*A*_3_ *B*_2_ *C*_1_ *D*_2_		—	

**Table 5:** Analysis of variance of the effects of several factors on CRW fermentation process.

Factor	Deviation sum of squares	Degree of freedom	*F* critical value	*F*-ratio	Significance
*A*	4.5513	2	2.2756	17.1394	<0.05
*B*	0.0514	2	0.0257	0.1935	ns
*C*	3.0627	2	1.5314	11.5338	<0.05
*D*	2.1452	2	1.0726	8.0787	<0.05

**Table 6:** Effects of dietary fermented chicory root waste on performance, feed utilization, and body index of *Cyprinus carpio* L. fed with high-fat diets.

Parameters	CG	HF	HF-L	HF-H
IBW (g/fish)	30.12 ± 0.29	30.06 ± 0.10	29.99 ± 0.04	29.97 ± 0.07
FBW (g/fish)	84.48 ± 1.46^b^	66.60 ± 0.95^c^	82.07 ± 1.64^b^	90.21 ± 1.13^a^
WGR (%)	180.53 ± 5.36^b^	121.61 ± 3.88^c^	173.70 ± 5.07^b^	200.95 ± 3.04^a^
SGR (%/day)	1.84 ± 0.03^b^	1.42 ± 0.03^c^	1.79 ± 0.03^b^	1.96 ± 0.02^a^
FCR (g/g)	1.50 ± 0.03^b^	2.00 ± 0.06^a^	1.61 ± 0.05^b^	1.48 ± 0.03^b^
*K* (g/cm^3^)	2.41 ± 0.03	2.34 ± 0.02	2.34 ± 0.04	2.35 ± 0.04
VSI (%)	9.36 ± 0.46^a^	8.37 ± 0.29^ab^	7.54 ± 0.49^b^	8.95 ± 0.61^ab^
HSI (%)	1.10 ± 0.04^bc^	1.30 ± 0.07^a^	1.19 ± 0.02^ab^	0.96 ± 0.04^c^

*Note:* The value is the mean ± SEM (*n* = 15). Different letters in the values showed a significant differences (*p* < 0.05), and the same or unmarked letters showed no significant differences (*p* > 0.05).

Abbreviations: CF, condition factor; FBW, final body weight; FCR, feed conversion rate; HSI, hepatosomatic index; IBW, initial body weight; SGR, specific growth rate; VSI, viscerosomatic index; WGR, weight gain rate.

**Table 7:** Effects of dietary fermented chicory root waste on whole fish proximate composition (g/100 g wet weight) of *Cyprinus carpio* L. fed with high-fat diets.

Parameters	CG	HF	HF-L	HF-H
MOI (%)	74.61 ± 1.26	73.03 ± 1.28	75.73 ± 1.84	73.51 ± 0.72
CP (%)	31.94 ± 0.51^b^	29.34 ± 0.93^c^	32.22 ± 0.51^b^	35.13 ± 0.40^a^
CL (%)	11.64 ± 0.50^c^	17.65 ± 0.38^a^	17.45 ± 0.46^a^	14.01 ± 0.04^b^
Crude ash (%)	8.09 ± 0.23	7.77 ± 0.62	7.84 ± 0.29	8.12 ± 0.71
LCL(%)	6.36 ± 0.27^c^	8.40 ± 0.13^a^	7.78 ± 0.20^ab^	7.49 ± 0.26^b^

*Note:* Different letters in the values showed a significant difference (*p* < 0.05), and the same or unmarked letters showed no significant difference (*p* > 0.05).

Abbreviations: CL, crude lipid; CP, crude protein; LCL, liver crude lipid; MOI, moisture.

**Table 8:** Effects of dietary fermented chicory root waste on serum and liver lipid-related profile of *Cyprinus carpio* L. fed with high-fat diets.

Parameters	CG	HF	HF-L	HF-H
Serum	—	—	—	—
TG (mmol/L)	2.36 ± 0.06^b^	3.11 ± 0.11^a^	2.24 ± 0.13^b^	2.40 ± 0.12^b^
HDL-C (mmol/L)	1.37 ± 0.07^a^	1.16 ± 0.03^b^	1.36 ± 0.04^a^	1.19 ± 0.04^b^
LDL-C (mmol/L)	1.00 ± 0.03^c^	1.21 ± 0.06^a^	1.13 ± 0.01^ab^	1.10 ± 0.01^bc^
Liver	—	—	—	—
TG (mmol/L)	0.83 ± 0.06^b^	1.40 ± 0.13^a^	0.87 ± 0.03^b^	1.13 ± 0.13^ab^

*Note:* The value is the mean ± SEM (*n* = 9). Different letters in the values showed a significant difference(*p* < 0.05), and the same or unmarked letters showed no significant difference (*p* > 0.05).

Abbreviations: HDL-C, high density lipoprotein cholesterol; LDL-C, low density lipoprotein cholesterol; TG, triglyceride.

## Data Availability

The datasets used and/or analyzed during the current study are available from the corresponding author upon reasonable request.
